# Upregulation of *DR3* expression in CD4^+^ T cells promotes secretion of IL-17 in experimental autoimmune uveitis

**Published:** 2011-12-29

**Authors:** Tingyu Qin

**Affiliations:** Chongqing Medical University, The First Affiliated Hospital of Chongqing Medical University, Chongqing, China

## Abstract

**Purpose:**

This study investigated the role of death receptor 3 (*DR3*) in experimental autoimmune uveitis (EAU).

**Methods:**

EAU was induced in B10.RIII mice by subcutaneous injection of interphotoreceptor retinoid-binding protein (IRBP) 161–180 emulsified with complete Freund’s adjuvant and evaluated with clinical and histopathologic observation. Total protein of draining lymph nodes (DLNs) was extracted from the control, EAU, or recovery phase mice. CD4^+^ T cells were separated from lymphocytes with magnetic-assisted cell sorting. At the same time, some of the CD4^+^ T cells were cultured with or without recombinant TL1A (rTL1A, the DR3 ligand) for three days, and the supernatants were collected for the interleukin-17 (IL-17) test. *DR3* mRNA and protein levels in CD4^+^ T cells and the endogenous concentration of TL1A in mice DLNs were assessed with real-time PCR or western blotting. Levels of IL-17 in the supernatants were determined with enzyme-linked immunosorbent assay.

**Results:**

Histopathological and clinical data revealed severe intraocular inflammation in the immunized mice. The inflammation reached its peak on day 14 in EAU and had resolved in the recovery phase (weeks 4–5 or more after IRBP immunization). CD4^+ ^T cells obtained from EAU (day 7 or 14) had higher levels of *DR3* mRNA and protein expression compared with the control group treated with complete Freund’s adjuvant alone and the recovery group. However, the *DR3* mRNA and protein levels on day 21 in EAU were similar to those observed in the control and recovery groups. The endogenous levels of TL1A were upregulated in EAU, and decreased in the recovery phase mice. Adding rTL1A increased the production of IL-17 by CD4^+^ T cells isolated from mice DLNs. Moreover, the increased IL-17 levels in the culture supernatant of CD4^+^ T cells from EAU were much higher than those from the control and recovery phase mice. However, the effects on promoting IL-17 production in TL1A-stimulated CD4^+^ T cells were similar between the controland recovery groups.

**Conclusions:**

Our data suggest that *DR3* expression is induced during EAU and may be involved in the development of this disease, possibly by promoting IL-17 secretion.

## Introduction

Experimental autoimmune uveitis (EAU) is a T cell–mediated autoimmune disease that serves as a model for several human posterior uveitis [1], such as Behcet’s disease, Vogt-Koyanagi-Harada syndrome (VKH), birdshot retinochoroidopathy, and sympathetic ophthalmia [2,3]. EAU is induced in animals by the adoptive transfer of retinal antigen-specific T lymphocytes [4,5] between syngeneic rodents [6,7], or by immunization with retinal antigens, such as the soluble retinal antigen (S-antigen) and interphotoreceptor retinoid-binding protein (IRBP) [8-14]. In addition, patients with uveitis have serum auto-antibodies to retinal antigens, including S-Ag and IRBP [15,16], and EAU can be induced by immunizing animals with the retinal antigens known to elicit responses in lymphocytes isolated from patients with uveitis [17].

During EAU, the integrity of the blood-retinal barrier is compromised, and monocytes/macrophages and antigen-specific T lymphocytes infiltrating the retina cause tissue damage [18]. Researchers have generally believed that EAU is caused by interferon gamma (IFN-γ) mainly secreted by CD4^+^ T helper_1_ (Th1) lymphocytes [19-22]. Recently, evidence suggested that newly recognized interleukin (IL)-17, produced by T helper_IL-17_ cells (IL-17-producing CD4^+^ T cells, Th17), plays a crucial role in this autoimmune disease by stimulating the initial influx of leukocytes into target tissues and mediating the tissue inflammation [18,23-26].

Death receptor 3 (DR3, TNFRSF25, TRAMP, LARD) is a member of the death-domain-containing tumor necrosis factor superfamily (TNFSF) of receptors. DR3 is primarily expressed on T cells and is essential for the development of diverse T cell–mediated inflammatory diseases [27]. TL1A (TNFSF15), a new TNFSF member, is currently the only known ligand of DR3 [28,29]. TL1A was first identified as a protein expressed on human endothelial cells and upregulated in response to tumor necrosis factor-alpha (TNF-α) and IL-1α. [28] Subsequently, expression of TL1A by antigen presenting cells (APCs) [29], such as human tissue macrophages [30], FcγR-activated peripheral blood (PB) monocytes, and monocyte-derived dendritic cells (DCs), was demonstrated [30-34].

CD4^+^ T cell activation and differentiation need not only the recognition of the antigen-major histocompatibility complex (MHC) class II complex by the cognate T cell receptor (TCR) but also co-stimulatory signals [35]. Most of these signals belong to either the B7-type molecules that bind CD28-like immunoglobulin (Ig) superfamily receptors or the TNFSF ligands that engage their receptor counterparts in the TNFRSF [36,37]. Several lines of evidence point to a role for TL1A-DR3 binding in modulating CD4^+^ T cell activation [27]. For example, in vitro, under conditions of suboptimal anti-CD3/CD28 stimulation, TL1A interaction with DR3 increases IL-2-driven proliferation and IFN-γ and granulocyte-macrophage colony-stimulating factor (GM-CSF) production, and TL1A:DR3 interaction also synergizes with IL-12 and IL-18 in stimulating TCR-independent secretion of IFN-γ by human PB CD4^+^ T cells [38,39]. In vivo, DR3-TL1A signaling has been associated with several autoinflammatory conditions, including allergic asthma [40], experimental autoimmune encephalomyelitis (EAE) [27,35], experimental antigen-induced arthritis (AIA) [30], inflammatory bowel disease [41], and allergic lung inflammation [42]. In addition, mice lacking the *DR3* gene (DR3ko) exhibited a reduction in all histopathological hallmarks of EAE, allergic lung inflammation [27], and AIA [30]. Moreover, some researchers have discovered that TL1A-DR3 interaction regulates Th17 development and IL-17 production [35], which play an important role in many autoimmune diseases [23,24,43-48]. Therefore, DR3 could be an attractive therapeutic target for T cell–mediated autoimmune and allergic diseases.

Thus far, studies have shown that CD4^+^ T cells are essential for the development of EAU [7,49-51]. However, the regulation of DR3 expressed by CD4^+^ T cells or the effect of DR3 on IL-17 production has not been investigated in the EAU model. This study is the first to investigate the expression and function of DR3 in CD4^+^ T cells in EAU. We found that *DR3* mRNA and protein levels were elevated in EAU and that stimulation with the DR3 ligand, TL1A, upregulated the secretion of IL-17 from CD4^+^ T cells. These results indicate that *DR3* may play an important role in the development and maintenance of EAU.

## Methods

### Mice and immunization

B10.RIII mice (6–8 weeks of age) were purchased from Jackson Laboratories (Bar Harbor, ME) and were housed under standard (specific pathogen-free) conditions. All animals were treated according to the ARVO Statement for the Use of Animals in Ophthalmic and Vision Research. IRBP_161–180_ (SGIPYIISYLHPGNTILHVD) was synthesized by Shanghai Sangon Biologic Engineering Technology and Services Ltd. Complete Freund’s adjuvant (CFA) containing 1.0 mg/ml *Mycobacterium tuberculosis* and pertussis toxin (PTX) were obtained from Sigma-Aldrich Co. (St. Louis, MO). To induce EAU, mice (8–12 weeks of age) were immunized subcutaneously with a 200 µl emulsion containing 50 µg IRBP_161–180_ in CFA. PTX (1.0 µg) was concurrently injected intraperitoneally as an adjuvant [52]. Control groups of mice received an emulsion of 50 µl PBS and 150 µl CFA, which was injected subcutaneously. Each experimental group consisted of 6–8 mice.

### Clinical examination and histopathological evaluation

After the animals were immunized, they were observed with slit lamp microscopy and ophthalmoscopy starting on day 7 until day 28. Eyes were enucleated from the control, EAU, and recovery phase mice (weeks 4–5 or more after IRBP immunization), were fixed for 1 h in 4% buffered glutaraldehyde, and were then transferred to 10% buffered formaldehyde until processing. Fixed and dehydrated tissue was embedded in paraffin and 5- to 7-µm sections were stained using a standard hematoxylin and eosin (H and E) approach. The intensity of EAU was scored from 0 to 4 in a blinded fashion according to the histopathological grading system previously described for murine EAU [50,53].

### Purification of CD4+ T cells, cultivation, and medium collection

Lymphocytes were collected from mice by draining lymph nodes (DLNs, inguinal and iliac), and CD4^+^ T cells were isolated using a specialized kit (Miltenyi Biotec, Palo Alto, CA). Briefly, lymphocyte suspensions were incubated with CD4 MicroBeads to sort CD4^+^ T cells. Then T cells were incubated in Gibco RPMI 1640 medium (Invitrogen, Carlsbad, CA) with anti-CD3 (1 µg/ml) and anti-CD28 (1 µg/ml) (eBioscience, San Diego, CA), with or without recombinant TL1A (rTL1A, 100 ng/ml [29,54]; R and D Systems, Minneapolis, MN). Incubations were performed in a 24-well culture plate for 72 h at 37 °C under an atmosphere of 5% CO_2_. After incubation, the supernatant was collected and stored at −80 °C.

### Reverse transcription PCR (RT–PCR) and real-time PCR

Total RNA was extracted from CD4^+^ T cells that had been isolated from control, EAU, or recovery phase mice using the RNA extraction kit (RNeasy Mini Kit, Qiagen, Hilden, Germany). Two micrograms of total RNA from each sample were used for reverse transcription using the Superscript III Reverse Transcriptase system (Invitrogen). The following sequences of the *DR3* and glyceraldehyde 3-phosphate dehydrogenase (*GAPDH*) primers used for real-time PCR: 5′-GGG CTA TCC TGA TCT GTG CAT-3′ (forward primer, *DR3*), 5′-ATG CCA GAG GAG TTC CAA GAGT-3′ (reverse primer, *DR3*), 5′-GAG AAC TTT GGC ATT GTG G-3′ (forward primer, *GAPDH*), and 5′-ATG CAG GGA TGA TGT TCT G-3′ (reverse primer, *GAPDH*). The mRNA expression levels were normalized to *GAPDH*, which was used as a reference housekeeping gene. PCR analysis was conducted on the real-time fluorescence quantitative PCR system using SYBR Green PCR Master Mix (Qiagen) according to the manufacturer’s instructions.

### Western blotting analysis

Total protein of DLNs was extracted from the control, EAU, or recovery phase mice using a protein extraction kit (BioChain, Hayward, CA). CD4^+^ T cells were homogenized using an ultrasonicator (BANDELIN Electronic, Bberlin, Germany), and the protein lysates were prepared for western blotting (WB) analysis. Fifty micrograms of protein from each sample was subjected to sodium fodecyl sulfate–PAGE, and the separated proteins were then transferred to polyvinylidene fluoride membranes. The membranes were incubated with anti-mouse TL1A (rabbit polyclonal antibody, Abcam, Cambridge, MA), anti-mouse DR3 (rabbit polyclonal antibody, Santa Cruz Biotechnology, Santa Cruz, CA), or anti-β-actin (rabbit polyclonal antibody, Santa Cruz Biotechnology), followed by a secondary antibody (goat antirabbit IgG-HRP; Santa Cruz Biotechnology). Proteins were detected using the Phototope-HRP western blot detection system (Cell Signaling, Danvers, MA).

### Enzyme-linked immunosorbent assay

Levels of IL-17 in culture media were measured using a commercially available enzyme-linked immunosorbent assay (ELISA) kit (R and D Systems). The detection limit of the kit is 15 pg/ml.

### Statistical analysis

Data were expressed as mean±SD. The experimental groups were compared with Student *t* tests, assuming equal variances for all data. P values < 0.05 were considered to be statistically different.

## Results

### Induction of EAU

**Figure 1:**
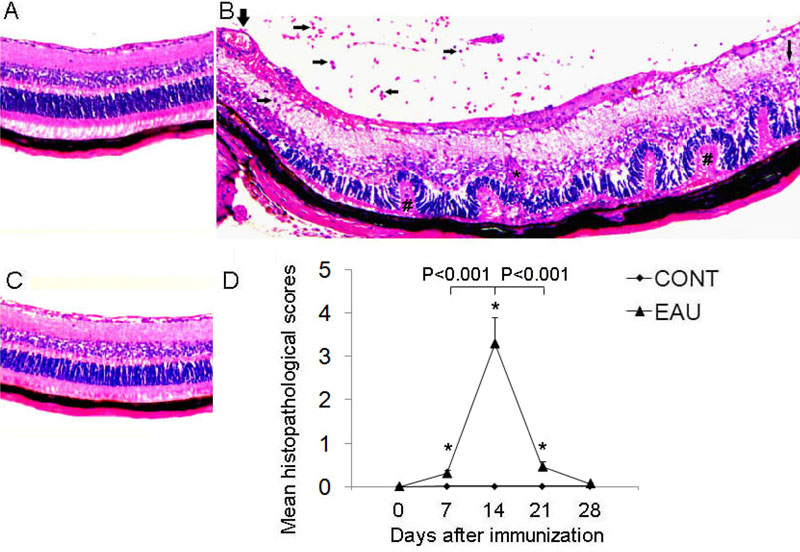
Histopathologic features of the eyes enucleated from experimental autoimmune uveitis (EAU), recovery phase and control mice. **A**: Eye of CFA controls. Normal retinal structure in a B10.RIII mouse. Hematoxylin-eosin (H and E) staining (magnification, 200×). **B**: Eye of EAU mice. An image from mice day 14 after IRBP immunization (at the peak of inflammation) shows inflammatory T lymphocytes (horizontal arrows) and macrophages (vertical thin arrow) infiltrating the vitreous and the retina, vasculitis (vertical bold arrow), damage to the retinal photoreceptor cell layer (pounds) and granuloma (asterisk). H and E staining (magnification, 200×). **C**: Eye of recovery phase mice. An image from mice week 4–5 or more after IRBP immunization shows no obvious inflammation in the retina. H and E staining (magnification, 200×). **D**: Mean histopathologic score during the development of EAU. * indicates p<0.001 when compared EAU with control mice. Each value represents the mean±SD (n=6).

EAU was successfully induced in B10.RIII mice after immunization with 50 µg IRBP_161–180_ in CFA [52]. Immunohistochemistry revealed severe inflammation in the posterior segment and a massive influx of inflammatory cells infiltrating the vitreous body and retina, vitritis, vasculitis, granuloma formation, and retinal photoreceptor lesions (Figure 1B). Inflammation was initially identified on days 7–9 after immunization and reached its peak by day 14. Inflammation was then followed by a rapid resolution and recovery (Figure 1D). As published reports have shown, there was no apparent inflammation in the control (treated with CFA only) [50,52] and recovery groups (week 4–5 or more after IRBP immunization) [53] (Figure 1A,C).

### Induction of EAU increases DR3 mRNA and protein expression in CD4^+^ T cells

**Figure 2:**
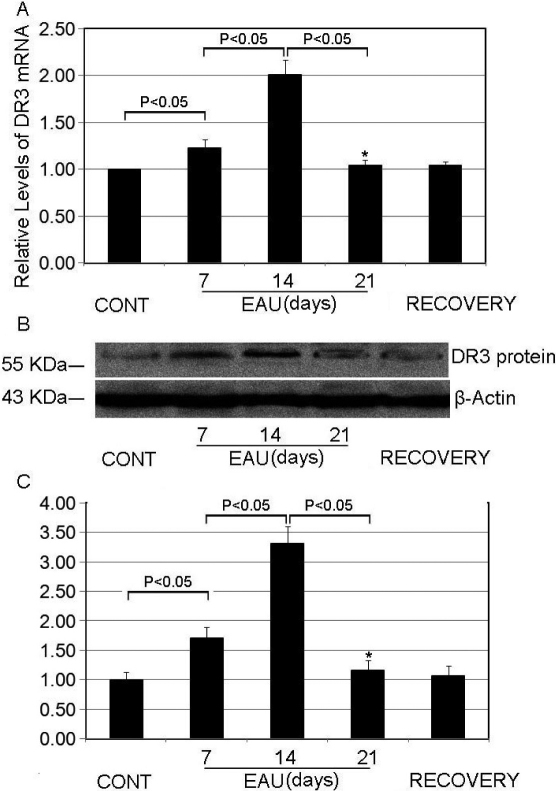
*DR3* mRNA and protein levels in CD4^+^ T cells from experimental autoimmune uveitis (EAU) increased. **A**: Real-time PCR analysis of *DR3* mRNA expression in CD4^+^ T cells isolated from the control, EAU, or recovery phase mice. *GAPDH* mRNA was used as a control to normalize the total mRNA levels. * indicates p<0.05 when compared day 7 with day 21 in the EAU group. **B**: western blotting analysis of DR3 protein expression in the CD4^+^ T cells. β-Actin was used as a loading control. **C**: Densitometry quantification of western-blotting results in panel **B**. * indicates p<0.05 when day 7 is compared with day 21 in the EAU group. Each value represents the mean±SD (n=6).

Since previous studies suggested that CD4^+^ T cells play a significant role in the development and maintenance of EAU [7,49], we used CD4 microbeads to sort CD4^+^ T cells from mouse lymphocytes obtained by draining LNs. The mRNA and total protein were extracted from CD4^+^ T cells for analysis with RT–PCR and WB. We found that the *DR3* mRNA and protein levels increased in CD4+ T cells from the EAU group 7 or 14 days after immunization (Figure 2). However, there were no obvious differences in *DR3* mRNA and protein levels between the controls, EAU (day 21), and recovery phase mice. We also found that the expression of *DR3* in CD4^+^ T cells in EAU achieved the highest level at day 14, and then declined (Figure 2). These data raise the question of the role of *DR3* upregulation in EAU.

### Secretion of IL-17 into CD4^+^ T cell culture medium is upregulated by the DR3 ligand, TL1A

**Figure 3:**
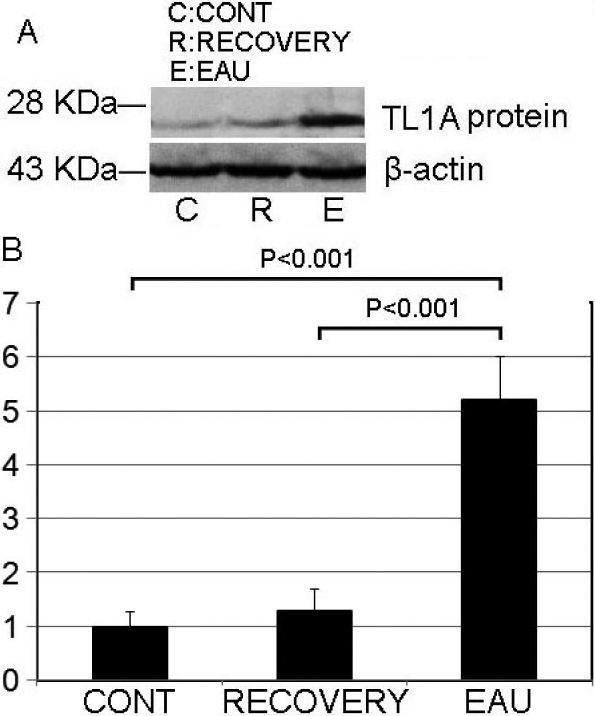
The mice endogenous level of TL1A was upregulated in experimental autoimmune uveitis (EAU). **A**: The concentration of TL1A in draining lymph nodes was detected with western blotting. **B**: Densitometry quantification of western-blotting results in panel **A**. Each value represents the mean±SD (n=6).

**Figure 4:**
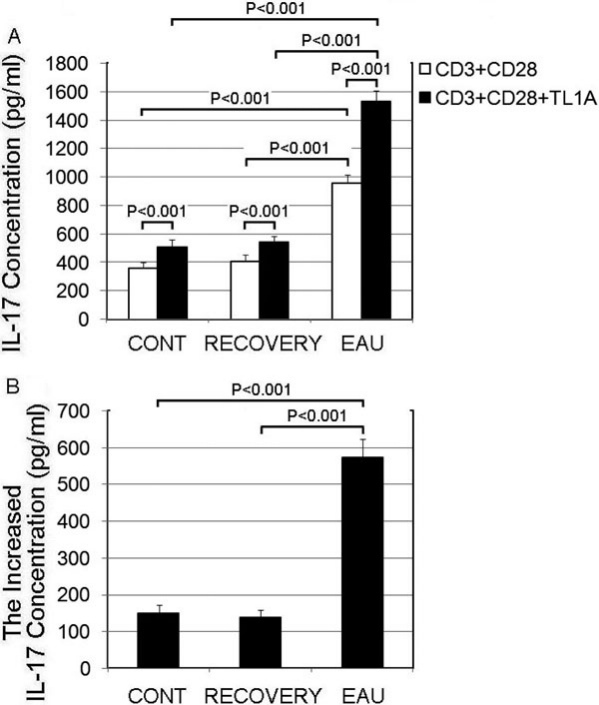
TL1A (TNFSF15) promoted secretion of IL-17 by CD4^+^ T cells. **A**: CD4^+^ T cells from the control, experimental autoimmune uveitis (EAU), and recovery phase mice were cultured in the presence of anti-CD3 (1 µg/ml) and anti-CD28 (1 µg/ml), with or without recombinant TL1A (100 ng/ml) for 72 h. IL-17 levels in the media were then determined with ELISA. **B**: The increased concentration of IL-17 secreted by CD4+ T cell in each group after TL1A stimulation for 72 h. Each value represents the mean±SD (n=6).

To determine the potential role for increased *DR3* expression in EAU, we chose the cells of the most severe inflammatory mice (day 14) to represent the EAU [53] as shown in Figure 1 and Figure 2. At first, we detected the endogenous levels of TL1A in mice DLNs with WB and found that the concentration of TL1A in EAU was upregulated; then it decreased in the recovery phase mice (Figure 3). After that, we added recombinant TL1A into the medium of the CD4^+^ T cells. Since under conditions of suboptimal anti-CD3/CD28 stimulation CD4^+^ T cells are thoroughly activated [35], we included anti-CD3/CD28 in the medium. We then measured IL-17 in the culture supernatants with ELISA and found that TL1A interaction with DR3 clearly promoted IL-17 production by CD4^+^ T cells compared with the media without TL1A in the control, EAU, or recovery mice (Figure 4A). This effect was significantly more pronounced in CD4^+^ T cells obtained from the DLNs of the EAU mice. Then we calculated the increased concentration of IL-17 after stimulated by TL1A for 2 h, and found that the elevated IL-17 levels in the culture supernatant in the EAU group were much higher, about fourfold, than that in the other two groups (Figure 4B). However, the increased levels of IL-17 stimulated by TL1A in the control group were similar to those in the recovery group (Figure 4B).

## Discussion

Uveitis is considered a typical T-cell mediating, organ-specific, autoimmune disease. EAU in B10.RIII mice induced by IRBP_161–180_, the best-studied model of uveitis recently shown to be IL-17 driven [23,44,55], has commonly been described as a monophasic disease [56], with a clinical peak about 2 weeks after immunization [37,57,58], then followed by remission and tolerance to reinduction; namely, EAU is a self-limited disease [1]. Similar to EAU, human posterior uveitis is also characterized by a bilateral granuloma, vasculitis, retinal lesions, and so on; however, human posterior uveitis often follows a relapsing and remitting course [59-61], of which the etiology and pathology are still elusive [62].

Previous studies showed that *DR3* is expressed on T cells [28], and DR3:TL1A signaling has been associated with several autoinflammatory diseases, including EAE, lung inflammation [27], Crohn’s disease [29], and experimental arthritis [30,63].

To further investigate its function, we analyzed the role of *DR3* in EAU. First, we examined the expression of *DR3* in mice. The *DR3* mRNA and protein levels in the CD4^+^ T cells of EAU mice were upregulated compared with the recovery phase mice and controls. Since DR3 expression coincides with the rapid induction of its ligand (TL1A) expression [29,32] and DR3 binding to TL1A regulates T-cell activation and expansion [27,63], we examined whether TL1A was also elevated in EAU. Results showed that the endogenous TL1A levels were significantly increased in EAU compared with the recovery phase mice and controls. These data imply that DR3 needs to be coupled with its ligand TL1A to execute its function.

Several studies show that Th17 cells or IL-17 are associated with ocular inflammatory diseases such as uveitis [60,64,65] and CD4^+^ T cells are capable of producing IL-17 [12]. Therefore, we studied the effect of DR3 on IL-17 production in CD4+ T cells and try to explain the mechanism of DR3 function in EAU. We found that DR3 interaction with TL1A could induce the increase of IL-17 production by CD4^+^ T cells. This is consistent with other findings that DR3-TL1A interaction regulates the Th17 cell function and IL-17-mediated autoimmune disease [35]. In summary, our study showed that increased DR3 production may be associated with the development of EAU in mice. These results indicate that stimulation of DR3 with TL1A could increase IL-17 production, with the suggestion that the DR3:TL1A signaling pathway may be involved in the pathogenesis of autoimmune uveitis.

In future research, we plan to determine how the interaction of TL1A with DR3 can increase the secretion of IL-17. Our studies will focus on elucidating cell signaling pathways that are activated in response to TL1A binding to DR3 and on the possibility of the production of cytokines other than IL-17. In addition, we want to knock out the DR3 gene in B10.RIII mice to observe whether the EAU model can still be induced. These studies will provide valuable new insights, and ultimately, we hope that elucidation of these mechanisms will enable the development of new therapeutic methods to treat human autoimmune and inflammatory diseases.
